# The Self-Propulsion of the Spherical Pt–SiO_2_ Janus Micro-Motor

**DOI:** 10.3390/mi8040123

**Published:** 2017-04-12

**Authors:** Jing Zhang, Xu Zheng, Haihang Cui, Zhanhua Silber-Li

**Affiliations:** 1 School of Environment and Municipal Engineering, Xi’an University of Architecture and Technology, Xi’an 710055, China; zhangjing102133@163.com (J.Z.); cuihaihang@xauat.edu.cn (H.C.); 2 State Key Laboratory of Nonlinear Mechanics, Institute of Mechanics, Chinese Academy of Sciences, Beijing 100190, China; lili@imech.ac.cn

**Keywords:** Janus micromotor, self-diffusiophoresis, bubble propulsion

## Abstract

The double-faced Janus micro-motor, which utilizes the heterogeneity between its two hemispheres to generate self-propulsion, has shown great potential in water cleaning, drug delivery in micro/nanofluidics, and provision of power for a novel micro-robot. In this paper, we focus on the self-propulsion of a platinum–silica (Pt–SiO_2_) spherical Janus micro-motor (JM), which is one of the simplest micro-motors, suspended in a hydrogen peroxide solution (H_2_O_2_). Due to the catalytic decomposition of H_2_O_2_ on the Pt side, the JM is propelled by the established concentration gradient known as diffusoiphoretic motion. Furthermore, as the JM size increases to O (10 μm), oxygen molecules nucleate on the Pt surface, forming microbubbles. In this case, a fast bubble propulsion is realized by the microbubble cavitation-induced jet flow. We systematically review the results of the above two distinct mechanisms: self-diffusiophoresis and microbubble propulsion. Their typical behaviors are demonstrated, based mainly on experimental observations. The theoretical description and the numerical approach are also introduced. We show that this tiny motor, though it has a very simple structure, relies on sophisticated physical principles and can be used to fulfill many novel functions.

## 1. Introduction

Motors that are designed to convert chemical or electromagnetic energy into mechanical energy are ubiquitous in people’s lives. In the last decade, micro/nano-motors have emerged, and they constitute a new field of technology attracting great interest from researchers. The pioneer work in micro/nano-motors involved a cylindrical micro/nano-motor using hydrogen peroxide (H_2_O_2_) as fuel, as proposed by Whitesides et al. [1] and the group of Sen and Mallouk [2]. Many other micro/nano-motors based on similar mechanisms were later fabricated [3,4,5]. These autonomous motors have two common features: (1) they have a metal-dielectric or bimetal double-faced structure, which is usually made of platinum–silica (Pt–SiO_2_) or platinum–gold (Pt–Au); and (2) they operate on the catalytic decomposition of H_2_O_2_, which is the energy source. As a result, these motors were named after the Greek double-faced god, Janus. The Janus micro-motor (JM) benefits from the heterogeneous structure that can spontaneously establish a local gradient around the JM. One obvious advantage is that JMs are self-motile due to the local gradient, and no external energy is required [6,7,8]. Encouraged by this advantage, researchers have developed many novel functions for Janus micro/nano-motors in recent years, such as drug delivery or ion detection in micro/nanofluidic chips [9,10,11,12,13,14,15], and purification of polluted water [15,16,17]. The JM has also shown wide application prospects as the power unit of a micro-robot [18].

**Figure 1:**
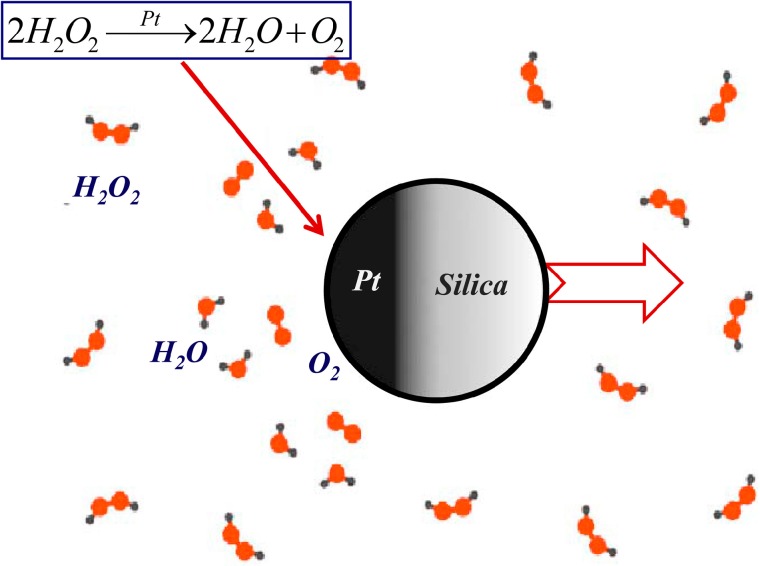
Schematic diagram of Pt–SiO_2_ spherical Janus micro-motor (JM) self-propulsion in H_2_O_2_ solution.

The concept of using the heterogeneous structure of a microparticle to build a local gradient field was proposed by the Nobel laureate P.G. de Gennes [19]. Basically, the JM’s self-propulsion is in the low Reynolds number (*Re*) flow [8,20], in which viscous effect is dominant. In this regime, in order to achieve propulsion, a microswimmer has to break time-reversal of the Stokes flow by employing moving units or deforming, as demonstrated by the scallop theorem [20,21,22]. A geometrically symmetric JM has to make use of the adjacent heterogeneoty to overcome the constraint of the scallop theorem [20,23,24]. Different surfaces with distinct physical/chemical properties can be used to build a heterogeneous concentration, temperature, or electrical field. Correspondingly, the self-motile motion of the microparticle driven by each field gradient is called self-diffusiophoresis [5,25], self-thermophoresis [26,27], or self-electrophoresis [28,29], respectively. Taking the Pt–SiO_2_ spherical micro-motor in H_2_O_2_ as an example, the concentration gradient is formed by the decomposition of H_2_O_2_ on the surface of the Pt hemisphere (Figure 1): 2H_2_O_2_ → 2H_2_O + O_2_. The higher molecular concentration on the Pt side provides the power to propel the JM in the other direction. Recalling the pioneer work of bubble propulsion based on microtubular engine developed by Mei and Solovev and their colleagues [30,31,32,33,34], the function of the JM could be significantly extended. It was recently found that O_2_ molecules could nucleate to form microbubbles if the size of the JM was up to O (10 μm) [35]. The capability to generate microbubbles by a JM, inspired by the work of the microtubular engine, could shed light on a novel, high-efficiency, form of fast propulsion. The mechanisms that how bubbles are generated and how bubbles propel the micromotors still need to be clarified. It is of great interest to thoroughly understand the mechanisms, including fluid–particle interaction and bubble dynamics.

Many experiments have been devoted to unveiling the physical mechanism behind a JM’s self-propulsion. A common consensus is that the motion of a JM whose size is smaller than 5 μm is dominated by self-diffusiophoresis, while that of a JM larger than 10 μm is primarily due to bubble propulsion [24,35,36]. However, some key issues remain unclear. For slow self-diffusiophoresis, from a statistical point of view, what is its typical behavior? What is the contribution of the rotational motion? For fast bubble propulsion, what is the exact force pushing the JM forward? How do we understand the bubble dynamics? In this review, we first organize the major experimental results to exhibit the big picture regarding a JM’s self-propulsion. By comparing with our results, we try to capture the typical behavior of the JM’s motion and explore the physics. Furthermore, we introduce both theoretical and numerical approaches to describe the self-propulsion of a Pt–SiO_2_ spherical JM. Finally, we discuss some important issues, such as improving the efficiency of JMs and manipulating them in real applications.

## 2. Self-Diffusiophoretic Motion

**Figure 2:**
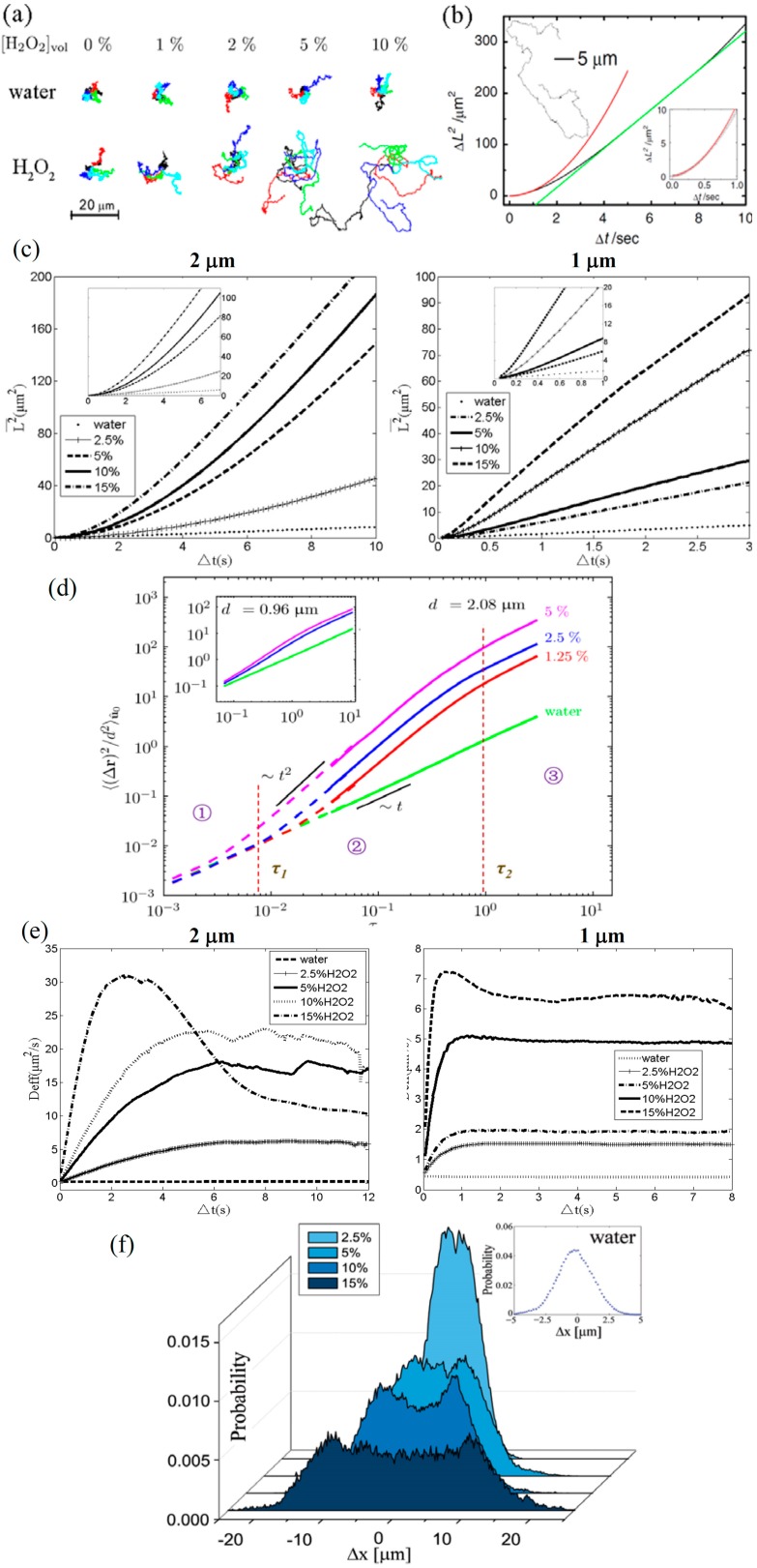
Characteristics of a JM’s translational diffusiophoresis. (**a**) The typical trajectory and (**b**) the time-varied mean square displacement (MSD) of JMs with a diameter of 1.6 μm [5]; (**c**) The typical MSDs of JMs with diameters of 2 μm and 1 μm, measured by our group; (**d**) The three-stage dimensionless MSD [37]; (**e**) The effective diffusion coefficient of JMs; (**f**) The double-peaked displacement probability distribution (DPD) [37]. Figure 2a,b is reproduced with permission from Howse et al. [5]; Published by APS, 2007. Figure 2d,f is reproduced with permission from Zheng et al. [37]; Published by APS, 2013.

The motions of JMs with diameters of 1–5 μm have been measured in some experiments [5,36,37,38]. The fabrication of a Pt–SiO_2_ JM usually gets help from e-beam evaporation to deposit a thin Pt layer on the hemisphere of a silica microsphere, for which details can be found in the literature [37,38,39] and the supporting information. As has been mentioned, these small JMs are propelled by the concentration gradient due to the catalytic reaction on the Pt side. This self-motile motion is called self-diffusiophoresis. The pioneer experiment of JM self-diffusiophoresis was performed by Howse et al. [5] using an optical microscope with the least observation time interval being about 10 ms. They showed the difference between self-diffusiophoresis and pure Brownian motion by comparing the trajectories of a JM in an H_2_O_2_ solution and pure water (Figure 2a) When the concentration of H_2_O_2_ was less than 1%, the trajectory of the JM was similar to that of Brownian random motion in pure water. With the increase of the H_2_O_2_ concentration, the self-diffusiophoretic trajectory exhibited the features of long-range directional movement, which was significantly different from the Brownian motion. Similar trajectories were reported in the literature [37,38,39,40,41,42,43]. The increased H_2_O_2_ concentration resulted in stronger catalytic reaction on the Pt surface. The chemical reaction kinetic was thus connected to the instantaneous speed of the JM movement. It was believed that the typical self-diffusiophoretic speed *V_DFP_* is linearly proportional to the reaction rate per unit area *k*, i.e., *V_DFP_ ~ k* [5,6]. In the experiment of Howse et al. [5], *k* was estimated to be about 5 × 10^10^ μm^−^^2^·s^−^^1^, which is consistent with our results.

The mean square displacement (MSD) of the JM’s self-diffusiophoresis in H_2_O_2_ solution was often measured to quantitatively illustrate the swimming characteristics. A particle-tracking method [43,44] was usually employed to obtain the MSD <*L*^2^> based on the trajectories, where <*L*^2^> denotes an ensemble average. A special image-processing technique was used to assure high-precision determination of the JM’s displacement. Figure 2b shows the typical MSD of JMs with a diameter *d* of 1.6 μm measured by Howse et al. [5]. Figure 2c shows the MSD of JMs with diameters of 1 or 2 μm measured by our group [37]. Different from the MSD of simple Brownian motion in water that increases linearly with time, the MSD of a JM’s self-diffusiophoresis in H_2_O_2_ solution shows nonlinear behavior at shorter times, and it turns linear at longer times. The parabolic-like MSD suggests a typical “ballistic motion” [45] driven by the concentration gradient. The transition time of the MSD is affected by the JM’s size. As shown in Figure 2c, the transition time of a 2-μm JM is approximately 5–6 s, while that of a 1-μm JM is only about 0.5 s.

We note that this transition time is close to the rotation characteristic time *τ_R_* = π*μd*^3^/*k_B_T* of a spherical JM (*μ* is viscosity, *k_B_* is Boltzmann constant, and T is temperature) [46]. Thus, the MSD results can be non-dimensionalized as shown in Figure 2d. The vertical axis is <*L*^2^>/*d*^2^, and the horizontal axis is *τ* = *t*/*τ_R_*. A green line with a constant slope of 1 is drawn to show the linear Brownian behavior. The dimensionless results show a three-stage behavior of the JM’s self-diffusiophoresis [37]: (1) At very short times *τ* < 10^−2^, the curve of the dimensionless MSD is similar to that of linear Brownian motion in pure water. This indicates that Brownian motion still dominates the JM’s motion because the concentration gradient has not yet been established. This stage is usually less than 10 ms, which was too short to be noticed in most previous experiments; (2) At intermediate times *τ* = 10^−2^–1, the curves of dimensionless MSDs all exhibit a slope of about 2. This slope is a typical signal of the ballistic motion driven by a concentration gradient, which is also called super-diffusive [5,46]; (3) The long-time stage begins at *τ* = 1, which is also *t* = *τ_R_*. It is interesting to see that the slope of the MSD returns to 1, which gives the name “Brownian-like motion” to this stage. However, it is obvious that the motion of the JM in the third stage is different from Brownian motion. It is the rotational motion that varies the directional propulsion in the second stage and decreases the slope back to 1. The above results reveal that the three stages of motion of the JM are dominated by different scales of physical effects.

The effective diffusion coefficient *D_eff_* is introduced to describe the self-diffusiophoresis of the JM. We can calculate *D_eff_* based on *D_eff_* = <*L*^2^>/4*t*, and the results are shown in Figure 2e. In pure water, the *D_eff_* is approximately constant. The *D_eff_* of a 2-μm JM is 0.20 μm^2^/s, and that of a 1-μm JM is 0.43 μm^2^/s; these are consistent with the results calculated by the Stokes–Einstein equation *D* = *k_B_T*/6π*μd*. In H_2_O_2_ solutions with different concentrations, *D_eff_* first increases rapidly and linearly with time, and then reaches an approximately constant value. The plateau values of *D_eff_* of 2-μm and 1-μm JMs are about 10–30 μm^2^/s and 1.5–7.2 μm^2^/s, respectively in 2.5%–15% H_2_O_2_ solutions. These values are increased by 1–2 orders of magnitude compared to that of simple Brownian motion in pure water. This shows the great potential of using super-diffusive microparticles in industry. From the Langevin equation, it has been deduced that a JM’s *D_eff_* can be written as [5,25]:

(1) $$D_{eff}=\frac{4R^{2}}{3\tau_{R}}+\frac{1}{4}{V_{DFP}}^{2}\tau_{R}+\frac{V^{2}{\tau_{R}}^{2}}{8t}(e^{-2t/\tau_{R}}-1),$$

where *V_DFP_* is the typical self-diffusiophoretic velocity of the JM. On the right side of Equation (1), the first term is the contribution of pure Brownian motion described by the Stokes–Einstein equation, the second term represents the contribution of the translational ballistic motion of self-diffusiophoresis, and the third term is the contribution of the rotation. It shows that the rotational time *τ_R_* is an important time scale in the dimensionless law of Figure 2d.

It is of great interest to investigate the statistical behavior of a JM’s self-diffusiophoresis [37,45]. The nonlinear behavior mentioned above should result in non-Gaussian behavior that is distinct from linear Brownian motion. The Displacement probability distribution (DPD) is measured in different H_2_O_2_ solutions to demonstrate the non-Gaussian behavior (Figure 2f). For Brownian motion in pure water, the DPD is consistent with the Gaussian distribution (the subplot in the top-right corner of Figure 2f). However, in different H_2_O_2_ solutions, every measured DPD exhibits a non-Gaussian double-peaked distribution. This double-peaked structure becomes more obvious with increasing concentrations of H_2_O_2_. The double peaks represent the most probable displacements of the JM’s self-diffusiophoresis motion, so they are of significance in controlling a JM’s motion. More interestingly, the non-Gaussian double-peaked structures are still evident even in the long-time stage (*t* > *τ_R_*), although the MSD has reverted to linearity. Further quantitative analysis of the non-Gaussian behavior relies on kurtosis, which is defined based on the fourth moment of the displacement. The kurtosis is negative for self-diffusiophoresis, as shown in our previous result [37]. More details of the statistical characteristics of the JM’s self-propulsion can be found in the literature [41,42,47,48].

**Figure 3:**
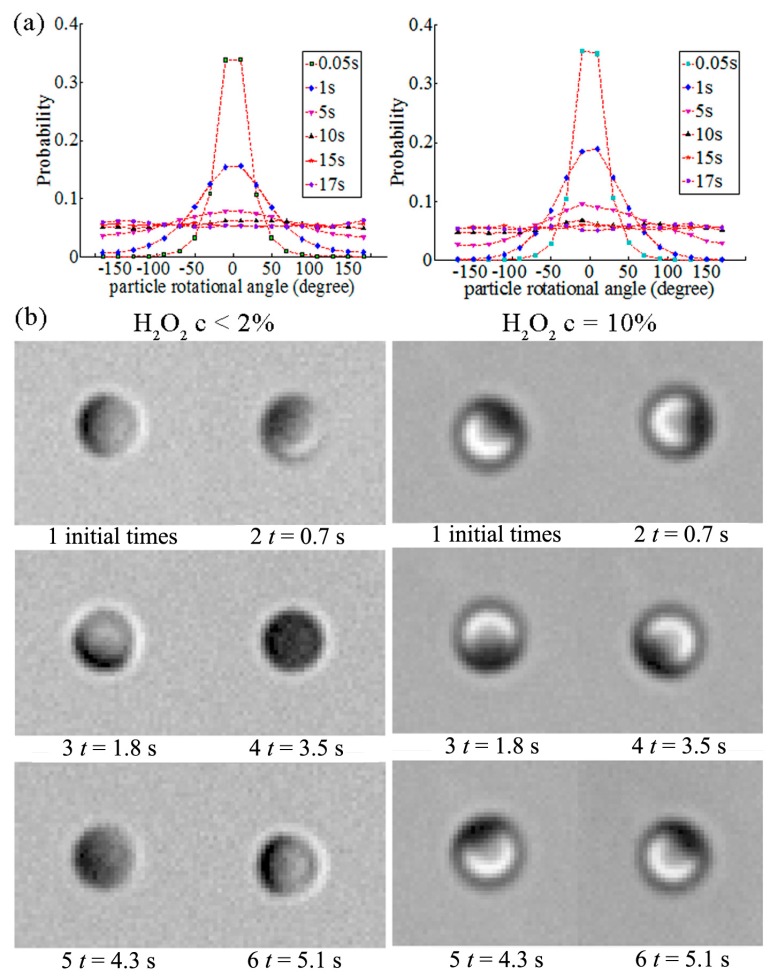
Rotational characteristics of the JM [43]. (**a**) A comparison between the rotational angle probability distribution of a JM with a diameter of 2 μm in different H_2_O_2_ solutions; (**b**) The JM exhibits 3-D rotation in an H_2_O_2_ solution with low concentration, while it exhibits 2-D behavior in an H_2_O_2_ solution with high concentration. Figure 3 is reproduced with permission from Zheng et al. [43]; Published by Springer, 2015.

The origin of the JM’s rotational motion is a topic that requires clarification. Figure 3a shows the rotational angle probability distribution of a JM in water and in 10% H_2_O_2_ solution, respectively. For Brownian motion in water, when *t* = 0.05 s, the rotational angle of the JM is mainly between −40° and 40°, and the peak of the probability distribution is 0.34. Then, the distribution turns flat. At *t* = 15 s, the probability distribution is almost uniform in all directions. The results in different H_2_O_2_ solutions are approximately the same as those in water. This indicates that the concentration gradient does not produce torque to affect the JM’s rotation. The only effect dominating the JM’s rotation is Brownian torque. The theory of Brownian rotation has been well established, with a typical time scale *τ_R_*. To control the rotation of the JM, ideas based on designing special geometrical shapes have been proposed. For example, “L-type” or boomerang-like micro-motors were fabricated to produce spiral motion [49].

The interaction between a wall and a JM is also interesting. The orientation and rotation of the Janus microsphere were observed to be influenced by the wall in our previous experiment [43]. Figure 3b illustrates the difference between a 3-D rotation in low-concentration H_2_O_2_ solution (<2%) and a 2-D rotation in the horizontal plane in high-concentration H_2_O_2_ solution (10%). This phenomenon has also been observed in a recent experiment by Das et al. [50]. The wall confinement was used to control the orientation and trajectory of the JM, as reported by Simmchen et al. [51]. The change of the JM’s direction near the wall is influenced by the symmetry-breaking of both the concentration and velocity fields. The confined self-diffusiophoresis of JMs has been a topic of great interest recently [52,53].

In addition, it should be noted that background flow has seldom been involved in previous experiments. In practical application, the self-diffusiophoresis of JMs should be coupled with surrounding fluid flow. Because the flow near the JM’s surface, the concentration gradient, and even the rotation of the JM is influenced by the background convective flow, the behavior of the JM may be changed significantly [54,55,56]. For example, Zottl and Stark reported that helical motion of a JM occurs in a microchannel with a square cross-section [54]. Due to the complexity of an analytic solution, there is still no consensus on this issue, and experimental research is urgently needed.

## 3. Fast Microbubble Propulsion

Following the microtubular engine, spherical JMs can also achieve fast microbubble propulsion. When the diameter of a spherical JM is larger than 10 μm, it is observed that the oxygen molecules generated by the decomposition of H_2_O_2_ can nucleate to form microbubbles. During this time, the self-propelled motion of the Janus microsphere is driven by the microbubbles. The mechanism of bubble nucleation remains unclear. It is believed that the curvature of the microsphere surface must be small enough [57]. In this section, we focus on the microbubble propulsion of a JM whose diameter ranges from 20 to 50 μm. These larger JMs move much faster than the smaller JMs described in the last section. This breaks the constraint that the phoretic speed of a JM is proportional to 1/*d* [58], which sheds light on the development of a faster JM. Obviously, the dynamics of the microbubble play an important role in the JM’s propulsion, although it is still under debate [24,59].

**Figure 4:**
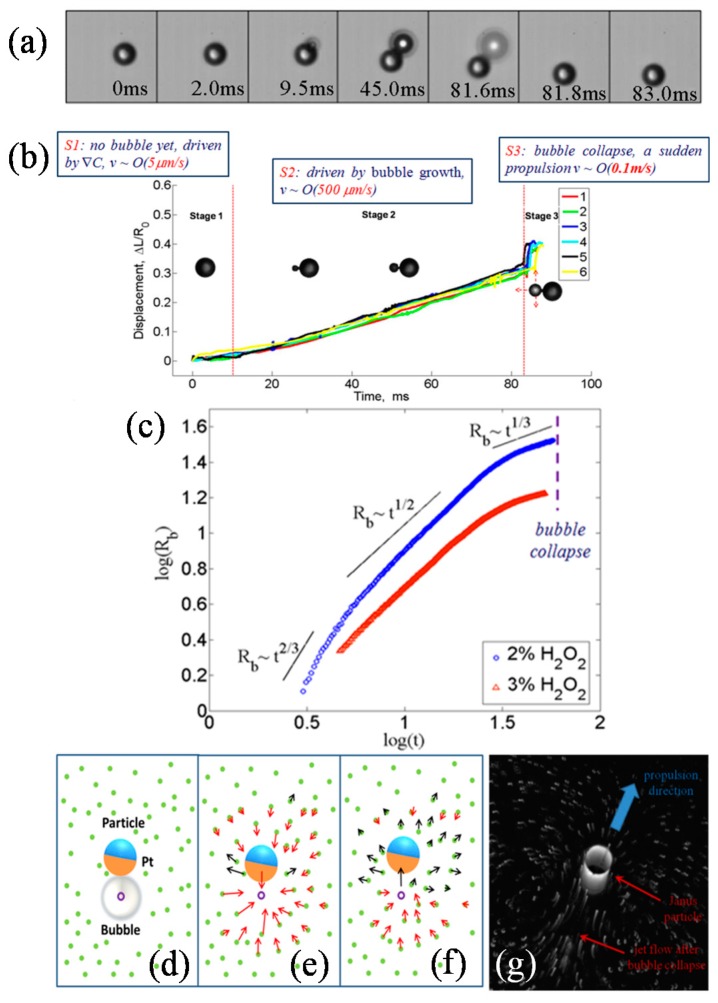
The JM’s motion propelled by a microbubble. (**a**) Image series of a JM’s motion in one bubble period which lasts about 83 ms; (**b**) Three-stage behavior of microbubble propulsion; (**c**) Different scaling laws during microbubble growth; (**d**–**f**) Variation of the flow field after microbubble collapse; (**g**) The microjet and wake vortices behind the JM shown by tracers. *R_b_*: microbubble radius.

We will first show the characteristics of the JM’s motion propelled by the microbubble. Figure 4a shows the image sequences of a JM with a diameter of 33.2 μm in a microbubble growth-collapse period. Just one bubble is generated throughout the period, and this is connected to the Pt surface of the JM by point-contact. The maximum bubble diameter is about 1.4 times larger than the JM. The complete cycle of one bubble growth–collapse period lasts about 83.0 ms. There is no visible microbubble at the Pt side of the JM until *t* > 9.0 ms. At *t* ≈ 9.5 ms, a bubble appears at the Pt side and grows gradually. At *t* = 45.0 ms, the diameter of the bubble becomes as large as that of the JM. The bubble’s diameter reaches its maximum value at *t* = 81.6 ms. The bubble suddenly collapses at about *t* = 81.8 ms. The collapse only takes about 10 μs according to our observation using a high-speed camera. At *t* = 83.0 ms, the JM’s propulsion stops due to the viscous effect, and the cycle is over. It is interesting to note that the H_2_O_2_ concentration does not influence the bubble growth significantly. The dominant factor of bubble growth is believed to be surface tension rather than chemical reaction rate. An early model suggested that the mechanism of bubble propulsion is the impulse produced by bubble disengagement [59], which was extended by Li et al. to bubble-propelled micro-motors with different shapes [60]. The model could explain the propulsion mechanism of microtubular motors studied by Mei and Solovev et al. [30,31,32,33,61]. However, it is not suitable for explaining the observation in our experiment that the bubble maintains direct contact with the JM directly.

Figure 4b shows the displacement of the same JM in six consecutive periods. Three stages in each period can be observed: (1) In the first stage (S1), no visible bubble is generated, and the JM’s typical speed is less than 10 μm/s. In S1, the motion of the JM is mainly affected by the concentration gradient, resulting in a low diffusiophoretic speed; (2) In the second stage (S2), the JM’s displacement increases almost linearly accompanying the bubble growth. The motion of the JM is propelled by the bubble growth force *F*_bubble_, and the typical speed is about 500 μm/s; (3) In the third stage (S3), the bubble collapses and the JM is pushed forward instantaneously. The speed of the JM is up to about 0.1 m/s in S3, resulting in a high *Re* number (*Re* ~ 10) in micro flow. However, we must emphasize that there is a back-pull phenomenon between S2 and S3. Before the strong forward motion, the JM sometimes withdraws after the bubble collapses. Manjare et al. [35] proposed that the drag from the bubble caused this back-pull, which is known as quasi-oscillatory motion. Below we will provide an explanation based on fluid mechanics.

We further analyze the bubble growth process. Figure 4c shows the growth of the microbubble radius *R_b_* with time *t* in 2% and 3% H_2_O_2_ solutions, respectively. One can see that there are different scaling laws during bubble-growth: at short times *R_b_* ~ *t*^2/3^, at intermediate times *R_b_* ~ *t*^1/2^, and at the end near bubble collapse *R_b_* ~ *t*^1/3^. The bubble-growth process is generally described by the Rayleigh–Plesset (R–P) equation [35,62]:

(2) $$P_{b}-P_{\infty }=\frac{2\sigma }{R_{b}}+\frac{4\mu }{R_{b}}{\dot{R}}_{b}+\rho (R_{b}{\ddot{R}}_{b}+\frac{3}{2}{\dot{R}}_{b}^{2}),$$

where *P_b_* and *P_∞_* are the bubble internal pressure and fluid pressure very far from the bubble, respectively. It is assumed that the bubble radius increases exponentially with time, *R_b_* ~ *t^m^*. Based on the ideal gas law *PV* = *nR_g_T* (the bubble volume is *V* = 4π*R_b_*^3^/3, the oxygen molar number is *n* = 2π*R*^2^*kt*, and *k* is a constant reaction rate), and neglecting high-order terms, the following three scaling laws are derived:

1. At short times, the microbubble radius *R_b_* is very small and the bubble pressure is dominated by the viscous term, $P_{b}\sim 4\mu {\dot{R}}_{b}/R_{b}$. Thus *R_b_* ~ *t*^2/3^ is derived.
2. At intermediate times, the bubble radius *R_b_* is about 10 μm and the bubble pressure is dominated by the surface tension, *P_b_* ~ 2*σ*/*R_b_*. Then, *R_b_* ~ *t*^1/2^ is obtained.
3. At the end, the bubble radius *R_b_* is quite large and the bubble pressure is close to the adjacent fluid pressure, *P_b_* ~ *P_∞_*. Then, *R_b_* ~ *t*^1/3^ is derived.

Finally, bubble collapse occurs when the bubble reaches its maximum size. A balance *P_b_* ~ *P_∞_* is reached, however, it will not be sustained because the oxygen supply becomes insufficient for the bubble with maximum size. The bubble suddenly shrinks due to *P_b_* < *P_∞_*, and the high order term in Equation (2) becomes negative. The theory of bubble cavitation is usually employed to describe this process, as mentioned in [35,62,63].

The above three scaling laws are in good agreement with experimental results, indicating there are three physical mechanisms dominating each stage of bubble growth. A previous study [36] used only a single power law, similar to *R_b_* ~ *t*^1/2^, to describe the bubble growth. Obviously, some physical mechanisms are missing in that description. In addition, although the R–P equation is derived based on the assumption of infinite unbounded fluids and uniform mass transfer through the bubble interface, we find that the R–P equation could still be used to approximately describe bubble dynamics near a JM.

Another key issue needing clarification is the origin of fast, instantaneous propulsion after bubble collapse. We used polystyrene (PS) micro-tracers suspended in the fluid to visualize the flow field. Figure 4d–f shows a series of schematic diagrams based on the real experimental video (see Supplementary Materials, Section 3) to illustrate the motions of the JM and the tracers. Figure 4d is the situation just before bubble collapse; Figure 4e,f is two successive frames after bubble collapse. The point “o” is the bubble center and the arrows represent the velocity vectors of the JM and the PS tracers. In the last moment before bubble collapse (Figure 4d), the bubble reaches its maximum size and maintains direct contact with the Pt side of the JM. In the first frame after bubble collapse (Figure 4e), the JM and the PS tracers around the bubble all move toward “o”. This can be described by the Stokes sink in fluid mechanics, around which the fluid velocity measured by the tracers is approximately *U*(*r*) ~ 1/*r*^2^ (*r* is the distance to the sink center “o”). This is why the JM withdraws at the beginning of S3, as mentioned above. In the second frame after bubble collapse (Figure 4f), the JM and the tracers in front of the microbubble are pushed forward and away from point “o”, while the tracers behind the bubble move toward “o”. This is due to the fact that the pressure near point “o” is much lower than that of the adjacent fluids (as described by the R–P equation). However, the existence of the JM whose mobility is much lower than that of the fluid molecules hinders the flow from the JM’s side. As a result, a jet appears flowing through point “o” and pointing to the JM. In fluid dynamics, this is known as the cavitation-induced-jet arising from the asymmetry of the medium around the bubble [63,64], as shown by Figure 4g. Considering the fact that the bubble and the JM usually do not locate in the same horizontal plane due to the difference of their density, the jet flow is not strictly in the horizontal plane. It is the horizontal component of the jet propelling the JM forward, as observed in Figure 4g. The instantaneous speed of the fluid could reach 1 m/s, about one order of magnitude larger than the maximum speed of the JM. At the same time, a pair of vortices appears behind the JM.

The above results show that the main source of the fast bubble propulsion is the horizontal component of the microjet produced by the bubble collapse rather than the impulse when the bubble leaves the surface of the JM. The impulse model proposes that the JM is propelled by the impulse of bubbles detaching from the JM’s surface. The experimental results shown in Figure 4 do not favor impulse mode, as the bubble never detaches from the JM. The microjet can focus the energy on propelling the JM, and therefore it can significantly improve the propulsion efficiency. This bubble cavitation-induced microjet provides a novel and important physical mechanism for propelling objects in micro-scale [35,65,66,67].

In addition, when the local concentration of micromotors is high, a large bubble emerges whose growth will significantly influence the collective motion of a group of JMs nearby [68]. The presence of the large bubble and the non-uniform temperature distribution along the liquid–gas interface will introduce a Marangoni flow near the bubble. This flow is stronger than the diffusiophoresis of the JM, and could be comparable to the bubble propulsion in some cases. The study of the collective motion of JMs is lacking, which could be applied in establishing self-assemble structures [69]. This new phenomenon provides an approach for manipulating collective motion of JMs based on bubbles.

## 4. Theoretical Description and Numerical Approach

Unlike the extensive experimental studies, theoretical or numerical approaches are not frequently seen in the literature. Nonetheless, they are helpful for their applications. The surface catalytic reaction, mass transfer process, Brownian motion, and even bubble dynamics are involved in the self-propelled motions mentioned above. The dimensionless numbers of self-propulsion reflect the basic physical characteristics. For the mass-transfer process, the Peclet number $Pe=V_{p}d_{p}/D_{o_{2}}$ ~ 10 is obtained for propulsion of larger JM bubbles, while *Pe* ~ 0.01 for smaller JM’s self-diffusiophoresis (*V_p_* is the self-propelled velocity, *d_p_* is the diameter of the JM, and $D_{o_{2}}$ is the diffusion coefficient of dissolved O_2_). This means that self-diffusiophoresis of smaller JMs is dominated by diffusion, while for the larger JMs, neither convection nor diffusion can be neglected. The Reynolds number *Re* is between 10^−6^ and 10^−3^, so self-propulsion of the JM is a low-*Re* flow. In this section, we will first introduce the theoretical description coupling the velocity and concentration fields, based on continuum equations. The chemical reaction flux and slip velocity on a JM’s surface are critical boundary conditions. As an alternative way, the Langevin equation can be used to describe the motion of the JM. The key issue in Langevin simulation is establishing the expression of the driving force due to the concentration gradient. Finally, for microbubble propulsion, the volume of fluid (VOF) method should be used to solve a JM’s motion and bubble dynamics, where the bubble is described by the phase-transition equation (see Supplementary Materials, Section 4).

### 4.1. Theoretical Description Based on Continuum Mechanics

The simplest theoretical description taking both velocity and concentration distribution into account was given by Anderson [8]. The key dynamic process was considered to occur in a very thin layer close to the liquid–solid surface, where an effective slip on the surface was proposed. The concentration equilibrium is reached as described by the Boltzmann distribution: *C* = *C_s_*exp(−*Φ*/*k_B_T*), where *C_s_* is the concentration at the surface and *Φ* is the potential energy. The momentum equations normal to the surface and along the surface are, respectively [8]:

(3) $$\frac{\partial p}{\partial y}+C\frac{d\Phi }{dy}=0$$

(4) $$\mu \frac{\partial^{2}u_{x}}{\partial y^{2}}-\frac{\partial p}{\partial x}=0$$

The typical velocity solved from the equations gives *u_x_* ~ *k_B_T*∇*C*/*μ*. Correspondingly, the phoretic speed of the JM is approximately *u* ~ ∇*C*, which can be also written as *u* ~ ∇*F* and is extended to other phoretic motions driven by a field *F* [6,8]. A detailed analysis about the scaling law of phoretic speed was given by Ebbens et al. [58]; this considered the JM size and the catalytic reaction activity.

For a complete numerical simulation, the low *Re* Stokes equation and the convection–diffusion equation should be coupled:

(5) ∇⋅U=0

(6) $$\mu \nabla^{2}U-\nabla p=0$$

(7) $$U\nabla C-D\nabla^{2}C=R_{dec}$$

The source term *R_dec_* in (7) is due to the decomposition of H_2_O_2_.

When the JM is far from the solid–liquid or gas–liquid interface, the simulation can be simplified to a 2-D symmetric problem. Relative coordinates are usually used: the JM is fixed and the fluid with a far-field velocity *V_p_* flows past the JM. The flow boundary condition on the surface of the JM is the slip boundary condition, *U_slip_* = *C_slip_*∇*C_t_* [6]. The flux boundary condition on the Pt surface is determined by the consumption flux of H_2_O_2_ that follows $f_{H_{2}O_{2}}$ = −*k_r_*$C_{H_{2}O_{2}}$, where the reaction rate *k_r_* is about 2.5 × 10^−3^ m/s [40]. Thus, the production flux of O_2_ is given by $\;f_{dec}$ = 0.5*k_r_*$C_{H_{2}O_{2}}$. On the SiO_2_ side, a zero-flux condition is applied. To get the solution of the above equations, the viscous drag should be balanced by the diffusiophoretic force due to the concentration gradient.

The wall effect is crucial when the JM moves close to the substrate wall. The near-wall effect will slow the motion of the JM and greatly increase the drag on the one hand, and will break the symmetry of both the flow and concentration fields around the JM on the other hand. The asymmetric fields will produce a torque that changes the JM’s orientation. Thus, to fully demonstrate the JM’s motion near a wall, 3-D numerical simulation is required, in which the slip coefficient *σ*, the equilibrium position *δ*, and the orientation angle *φ* are three parameters to be solved for in the simulation. Accordingly, three equations about force or torque equilibrium are needed. For the force equilibrium, in the horizontal direction the diffusiophoretic force is balanced by the Stokes drag force: *F_Stokes-X_* + *F_DFP-X_* = 0, while in the vertical direction gravity should be involved: *F_Stokes-Y_* + *F_DFP-Y_* = *G*. Due to the symmetry of geometric and flow conditions, the torques in the *X*-axis and *Z*-axis are naturally balanced. For the torque equilibrium, as the flow field and concentration field are not symmetric, the torque generated by Stokes drag *T_Stokes_* should be balanced by the torque generated by diffusiophoretic force *T_DFP_*: *T_Stokes_* + *T_DFP_* = 0. It is worthwhile to note that all the forces and torques listed above are dependent on the three parameters *σ*, *δ*, and *φ*. Solutions based on this approach have been obtained [40], which could help us to understand the wall effects mentioned in previous literature.

### 4.2. Kinetic Motion Solved by Langevin Equation

When a JM’s size is small enough, the random thermal disturbance *R*(*t*) will become important enough to result in Brownian motion. In 1908, Langevin introduced *R*(*t*) as a random force into the Newton equation and established the Langevin equation of single particle motion. The effect of external fields can also be directly introduced into the Langevin equation to solve the motion of a JM under multiple physical fields. In the present case of self-diffusiophoresis due to concentration gradient, the Langevin equation is established as:

(8) $$m\frac{d^{2}x}{dt^{2}}=F_{Stokes}+F_{Brownian}+F_{DFP},$$

where *F_Brownian_* is a random force whose time-average value is zero, *F_Stokes_* = 6π*R_p_μV_p_* is the Stokes drag, *R_p_* is the radius of the JM, *V_p_* is the velocity of the JM, and *F_DFP_* is the self-diffusiophoretic force. *F_Brownian_* is produced by the impact of adjacent fluid molecules, and could be considered an equivalent force acting on the center of the JM:

(9) $$F_{Brownian}=\xi_{1}\sqrt{\frac{12\pi k_{B}T_{0}\mu R_{p}}{\Delta t}},$$

where *ξ*_1_ is a random number, *k_B_* is Boltzmann’s constant, *T*_0_ is the thermodynamic temperature, and Δ*t* is the observation time interval. Obviously, *F_Brownian_* decreases with an increasing time interval, and approaches zero for a long time interval. *F_DFP_* cannot be directly defined. However, it has been reported that *F_DFP_* is proportional to the drift velocity (*V_DFP_*) of a JM under pure self-diffusiophoresis [5,35]. Thus, $F_{DFP}=6\pi \mu R_{p}V_{DFP}\vec{\theta }$ is proposed, where $\vec{\theta }$ is the rotational angle of the JM pointed from the Pt side to the SiO_2_ side.

At any moment, $\vec{\theta }$ is still unknown due to the Brownian random torque. To solve the Langevin equation, the change of $\vec{\theta }$ due to Brownian torque must be introduced. The rotational angular velocity *Ω* is used to describe the change of the rotational angle:

(10) $$\Omega =\frac{d\vec{\theta }}{dt}=\frac{\Gamma_{\theta }}{f_{r}}=\frac{F_{Brownian}R_{p}}{8\pi \mu R_{p}^{3}}=\xi_{1}\sqrt{\frac{3k_{B}T_{0}}{16\pi \mu R_{p}^{3}\Delta t}},$$

where *Γ_θ_* is the torque and *f_r_* is the rotational friction coefficient of the viscous fluid. Combining the Langevin equation of translational motion and the rotational equation of *Ω*, the kinetic motion of a small JM can be fully solved. This approach can reveal the competition between Brownian motion and pure diffusiophoretic motion, which is hardly achieved by the methods based on continuum mechanics except for fluctuating hydrodynamics.

## 5. Discussion

### 5.1. Propulsion Efficiency

Improving the efficiency of the JM is crucial in application. Unfortunately, the energy transfer efficiencies *η* of existing JMs as reported in the literature are unsatisfactory [70,71]. The efficiency *η* is usually too low to be applied (10^−10^) [70], mainly because the kinetic energy of the JM will rapidly dissipate in a low *Re* viscous flow. We estimate the energy transfer efficiency of fast microbubble propulsion based on the energy variation of the system before and after bubble collapse. At the onset of bubble collapse, the bubble surface energy, defined by *E_b_* = 4π*R_b_*^2^*σ ~* 10^−9^ J, reaches its maximum value. Based on the observed speed *V_p_* of the JM after bubble collapse, the JM’s kinetic energy is estimated as *E_k_* = 2π*ρR_JC_*^3^*V_p_*^2^/3 ~ 10^−11^ J. Therefore, the energy efficiency *η* ~ *E_k_*/*E_b_* is about 1%. This efficiency is 7–8 orders of magnitude larger than that estimated by Wang et al. for phoretic JMs [70]. This high efficiency occurs because the bubble cavitation-induced microjet focuses the energy on propelling the JM forward rather than being transferred to the adjacent liquid.

### 5.2. Microfluidic Applications

**Figure 5:**
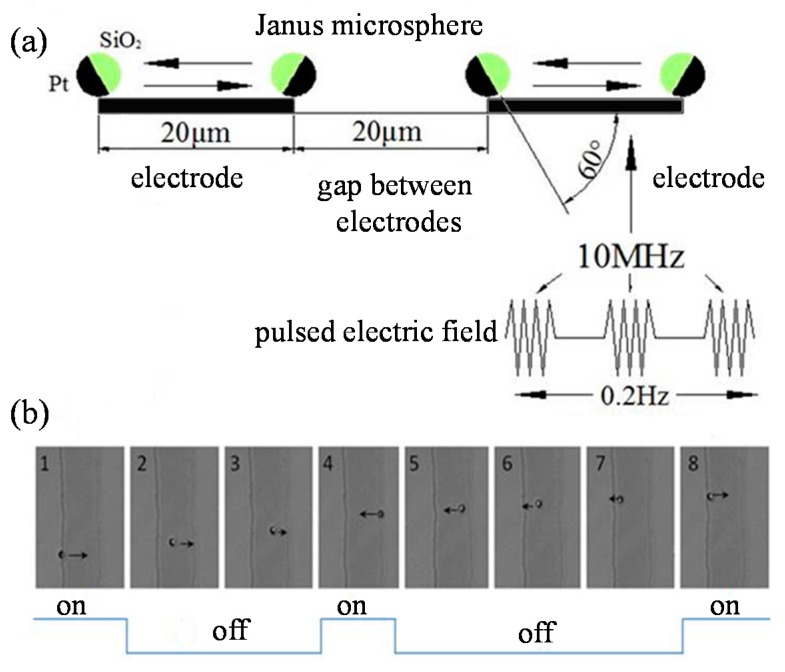
The back-and-forth motion of a “microshuttle” JM between different electrodes based on positive dielectrophoresis (pDEP) [73,74]. (**a**) The schematic diagram; and (**b**) the image series of a JM’s reciprocating microshuttle motion in one electrode. Figure 5 is reproduced with permission from Chen et al. [73]; Published by AIP, 2014.

Many studies have been devoted to realizing more and better functions of the JM [9,10,11,12]. A challenge of manipulating a JM in microfluidic application is to control its direction. For small JMs, since Brownian motion significantly influences both translational and rotational motion of the JM, new techniques should be developed to overcome the randomness. In this section, a microshuttle technique based on dielectrophoresis is introduced. The Pt–SiO_2_ type JM under high-frequency AC voltage always exhibits a positive dielectrophoresis (pDEP) response [72], and the JM is attracted to the electrode border, keeping its Pt side outward and its SiO_2_ side toward the electrode. In the experiment, JMs with a diameter of 2 μm are immersed in 5% H_2_O_2_ solution [73,74]. Then, a pulsed AC field (voltage 2 V, frequency 10 MHz, switching frequency 0.2 Hz, see Figure 5a) is applied to the stripe-like ITO electrodes (width 20 μm). The dielectrophoretic force will suppress the self-diffusiophoretic motion of the JM and trap the JM at the border of the electrode when the AC voltage is on (Figure 5b). The pDEP response of the JM disappears immediately after the AC voltage is turned off, and the self-propulsion becomes dominated. Because the orientation of the JM is toward the electrode, the JM will move along its orientation as long as it has not rotated too much in a short time. Since one “on–off” period is 5 s in the experiment, the self-propelled time of the JM is 2.5 s. Based on the typical self-diffusiophoretic speed (5–10 μm/s) mentioned in Section 2, the JM can move to the position near the opposite border of the electrode. When the AC voltage is on again, the JM will soon be trapped at the opposite border by the pDEP force and change its orientation (Figure 5b). When the AC voltage is off again, the JM will start to return to its original position. Thus, the JM’s motion in a microfluidic system can be manipulated like a microshuttle under an AC voltage with a suitable frequency [73,74]. By controlling the frequency of the AC voltage, the JM can also move back and forth between different electrodes.

## 6. Conclusions

In this paper, we introduce the mechanisms of two distinct self-propulsion methods of the spherical Pt–SiO_2_ JMs: self-diffusiophoresis and microbubble propulsion. Major experimental results and theoretical/numerical approaches are reviewed. The former motion occurs for JMs whose diameter is roughly smaller than 5–10 μm, and it results from the concentration gradient established by decomposition of H_2_O_2_ on the Pt surface. The MSD of the self-diffusiophoresis exhibits a three-stage behavior: simple Brownian motion at short times, ballistic motion at intermediate times, and Brownian-like motion at long times. The self-diffusiophoresis can be seen as a superposition of a translational ballistic motion and a Brownian rotation. The typical time scale is the rotational time of the microsphere *τ_R_*. The statistical characteristics of self-diffusiophoresis consist of the double-peaked structure of DPD and negative kurtosis. The latter motion occurs when the JM’s diameter is larger than 10 μm. In this case, the JM moves with a speed about 500 μm/s during microbubble growth, and surprisingly, its speed can reach 0.1 m/s during bubble collapse. The strong instantaneous propulsion originates from the bubble cavitation-induced microjet. This microjet can significantly improve the energy efficiency, and thus provides a novel power source for future micro-motors. It is found that the R–P equation can be approximately used to describe the bubble dynamics.

To develop novel JMs in the future, we still need to pay attention to several issues. First, the materials and functions of micro/nano-motors are rapidly developed. There are many new types of micro/nano-motors rather than the spherical Janus ones. Second, the commonly used JMs or other micromotors work in chemical solutions that could have toxicity. It is important to develop micro/nano-motors that can simply work in water or nontoxic solutions [11,75,76]. Third, the bio-compatibility of the materials of micro/nano-motors is also important for bio-medicine applications [77,78,79,80,81,82]. Fourth, developing multi-function and high-efficiency micro/nano-motors requires the involvement of other physical fields. JMs and other types of micro/nano-motors driven or controlled by ultrasound, electromagnetic field, and light pressure are now under extensive investigation [83,84,85,86,87,88]. A thorough understanding of the physics is very helpful for designing powerful micro/nano-motors.

## Supplementary Materials

The following are available online at www.mdpi.com/2072-666X/8/4/123/s1, Figure S1: Three successive images during bubble collapse.
