# Supplementary material for: The Self-Propulsion of the Spherical Pt–SiO2 Janus Micro-Motor
Source: Micromachines (Basel). 2017 Apr 12;8(4):123. doi: 10.3390/mi8040123 (PMC6189969; doi:10.3390/mi8040123)
Supplement: Supplementary file 1 [file micromachines-08-00123-s001.pdf]

Supplementary Materials: The Self-Propulsion of the Spherical Pt–SiO_2 Janus Micro-Motor

Jing Zhang, Xu Zheng, Haihang Cui, Zhanhua Silber-Li

1. Fabrication of the JMs

The Pt–SiO_2 Janus micromotors (JMs) were fabricated by covering a layer of Pt onto one hemisphere of SiO_2 microspheres. The silica particles with different diameters were produced by the University of Petroleum in China. To fabricate the JM, an aqueous suspension of silica particles is first deposited on a 4-inch silicon wafer by spin coating at low speed (800 rpm). After evaporating the water, a single layer of particles is formed on the wafer. Then, using electron beam evaporation (by an Innotec ebeam evaporator in the Institute of Semiconductors, Chinese Academy of Sciences), a layer of Pt (thickness about 10 nm) is deposited on the upper half surfaces of the particles. Finally, JMs were scratched from silicon wafer with a sharp blade and mixed with ultrapure water (18.2 MΩ·cm) to store. The treatment of ultrasonic oscillatory was applied to keep the monodisperse status of JMs prior to each experiment.

2. Experimental Methods

The experimental setup consisted of a Nikon ECLIPSE Ti-U inverted fluorescence microscope equipped with different objectives (20×, 40×, 100×). A high-speed CCD (charge-coupled device) camera (Phantom v7.3, Vision Research, Inc., Wayne, NJ, USA) was used to capture images. The capturing speed is up to 88000 fps and the corresponding exposure time is about 50 μs. The 30% H_2O_2 solution was mixed with JMs solution to obtain the desired concentration. A 50-μL droplet was dipped onto coverslip and rested for a few minutes to avoid the disturbance of flow. Then, the microscope was adjusted to position one JM to the center of view field and then started to record the motion of JM.

We analyzed the motion of JM and the evolution of the bubble from the video. The selected video was converted into a series of TIF images by IMAGEJ. We applied the following method to determine the exact center of the JMs, which appear half bright and half dark in the images. First, the find edge function of the program IMAGEJ was used to highlight the sharp intensity changes at the particle edges. This function offered a way to reconstruct the round shape of the JM. Secondly, using the Gaussian blur function of IMAGEJ, the gray scale value distribution in the JM domain was determined. The point with the maximum gray scale value was considered to be the center of the particle. This method has ±0.5 pixel accuracy. After this preprocessing, the JM positions (x,y) can be tracked by the software VIDEO SPOT TRACKER (V07.02). To guarantee that only individual JMs are tracked, we omitted aggregated JMs and used a "dead zone" function, by which the region of approximately one diameter around the JM was monitored. Therefore, JM aggregation as well as JM collisions and interactions can be excluded from our investigation. After obtaining the JM position in each frame from the video, the displacement, speed, as well as the MSD (mean square displacement) can be calculated easily.

In order to decide the size and position of microbubble, we also operated "finding edge" function in IMAGEJ to determine the edges of bubble. The geometric center was tracked by VIDEO SPOT TRACKER to collect the coordinate data at each frame. The time information was read out from the setting of fast CCD camera. Thus, we can calculate the size, the displacement and the velocity of bubble.

3. Observation of the Microjet

In order to visualize the flow field of the microjet, polystyrene tracers (Thermo Fisher Scientific) with diameter of 2 μm were suspended in the solutions. Figure S1 shows three successive images captured by high speed camera with 9900 fps. The time interval between each two frames is about

100 μs. The tracers' motion is tracked to present the results shown in Figure 4d–f. In Figure S1a, the microbubble can still be seen below the JM in the center. In Figure S1b, the bubble has collapsed and the adjacent tracers move toward the bubble center. An instantaneous forward displacement can be seen in Figure S1c. By overlapping the images during this process, the jet flow shown in Figure 4g can be clearly visualized.

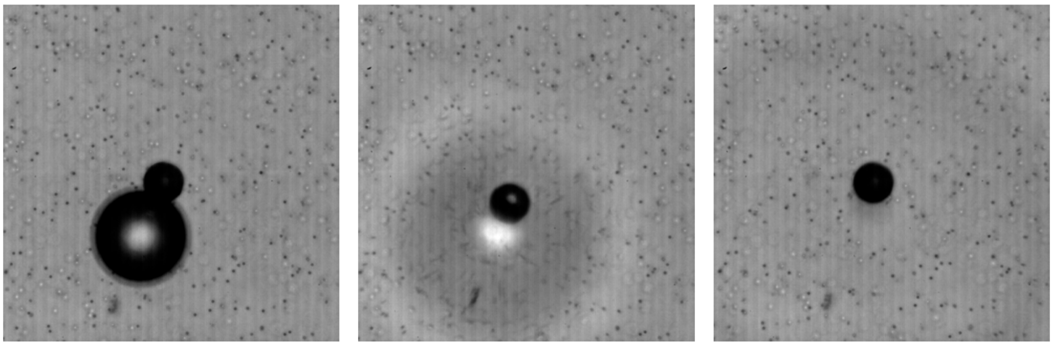

(**a**) (**b**) (**c**)

Figure S1. Three successive images during bubble collapse. Figure 4d–f are plotted based on the motions of the tracers and the JM observed in (**a**–**c**), respectively.

4. Volume of Fluid (VOF) Method

When the JM's size is larger than 10 μm, oxygen bubbles usually emerge. The propulsion mechanism due to the presence of bubbles is completely different from self-diffusiophoresis. The numerical method that is usually used to deal with two-phase flow is the VOF method. The governing equations of the VOF method contain the modified Navier–Stokes equations and a volume fraction equation:

$$\frac{\partial \rho}{\partial t} + \nabla \cdot (\rho U) = 0 \tag{S1}$$

$$\frac{\partial (\rho U)}{\partial t} + \nabla \cdot (\rho U U) = -\nabla p + \nabla \cdot (\mu \nabla U) + \sigma \kappa \tag{S2}$$

$$\frac{\partial \alpha}{\partial t} + (U \cdot \nabla)\alpha = 0 \tag{S3}$$

where σ is the surface tension coefficient, κ is the surface curvature, α is the volume fraction ($\alpha = 0$ indicates gas inside the bubble, $\alpha = 1$ is liquid, and $0 < \alpha < 1$ represents the interface region). There are several differences compared to the continuum approach of Equations (5)–(7) in the main text. First, the density variation of the compressible gas phase should be considered; the density in the bubble is not constant. Usually, the ideal gas law, $p = \rho RT/M$, should be applied to demonstrate the gas state in the bubble. Second, as observed in the experiments, the propelled speed could be very large, and *Re* is up to O (1–10). Thus, the momentum equation of the flow is the N–S equation instead of the Stokes equation of low Re flow. Third, the surface tension is treated as a force from the interface that appears in the momentum equation. Fourth, based on the scalar transport Equation (S3), the density and viscosity are defined as summations for the two phases, i.e., $\rho = \alpha_1\rho_1 + \alpha_2\rho_2$ and $\mu = \alpha_1\mu_1 + \alpha_2\mu_2$, respectively.

© 2017 by the authors. Submitted for possible open access publication under the terms and conditions of the Creative Commons Attribution (CC BY) license (http://creativecommons.org/licenses/by/4.0/).
